# Outcomes of a National Cohort of Children with Acute Severe Ulcerative Colitis

**DOI:** 10.3389/fped.2018.00048

**Published:** 2018-03-08

**Authors:** Abisoye O. Akintimehin, Ríoghnach Sinead O’Neill, Conor Ring, Tara Raftery, Séamus Hussey

**Affiliations:** ^1^National Centre for Paediatric Gastroenterology (NCPG), Our Lady’s Children’s Hospital, Crumlin (OLCHC), Dublin, Ireland; ^2^National Children’s Research Centre, Crumlin, Ireland; ^3^Royal College of Surgeons of Ireland, University College Dublin, Dublin, Ireland

**Keywords:** acute, severe, ulcerative, colitis, infliximab, children, paediatric, steroid

## Abstract

**Aim:**

All Irish children with ulcerative colitis (UC) attend the National Centre for Paediatric Gastroenterology at Our Lady’s Children’s Hospital, Crumlin. The aim of this study was to determine the outcomes of children with acute severe ulcerative colitis (ASC) and the impact of infliximab on these outcomes following its introduction for this indication in 2011.

**Methods:**

A retrospective chart review of all patients admitted with ASC between January 1, 2009 and December 31, 2015 was undertaken. Patients were identified from the departmental database cross-referenced with the hospital inpatient enquiry system. Inpatients with a paediatric ulcerative colitis activity index (PUCAI) of ≥65 were included. Data collected included baseline demographic and laboratory data, concomitant treatments, PUCAI scores on days 3 and 5, second-line treatments, surgery, and discharge outcomes. Infliximab dose, frequency, and available therapeutic drug monitoring results were recorded, along with clinical response outcomes (remission, primary, and secondary loss of response). The cohort was sub-analysed to determine if there was any era effect pre- and post-introduction of infliximab (2009–2010 and 2011–2015, respectively).

**Results:**

Fifty-five patients (M:F = 1.4:1) were treated for acute severe colitis over the study period (8 in the pre-infliximab and 47 in the post-infliximab era) and 46/55 (86%) had steroid-refractory disease. Of these, 7/8 (88%) required colectomy in the pre-infliximab era, compared with 15/47 (36%) in the post-infliximab era. The remission rate with second-line infliximab was 61% at maximal follow-up. There were no identifiable factors that predicted likely success or failure of infliximab, including gender, CRP, day-3 and day-5 PUCAI scores. Of the 33 patients treated with infliximab, dose increase was required in 23/33 (70%); 21/33 (64%) received an accelerated dose schedule, and 9/33 (27%) eventually needed colectomy. Primary and secondary loss of response to infliximab was seen in one and nine patients, respectively.

**Conclusion:**

This is the first population-based study of the outcomes of severe UC in Irish children, and suggests a higher burden of steroid-refractory disease compared with previous international studies. While infliximab treatment has led to reduction in colectomy rates, a significant proportion of patients lose therapeutic effect.

## Introduction

Ulcerative colitis (UC) is a chronic relapsing inflammatory disease of the colon affecting both adults and children that extends variably from the rectum to the caecum. The incidence of paediatric inflammatory bowel disease (IBD) in the UK and Ireland has been reported as 5.2 per 100,000 per year (1). The highest rates of UC internationally have been reported in northern Europe, Canada, Unites States, and Australia (2). While a recent systematic review showed an overall stable or decreasing incidence of IBD in North America and Europe, an increasing incidence has been reported in Africa, Asia, and South America (3). There is evidence of a global increase in the incidence of paediatric-onset IBD (4). While there are many similarities between adult-onset and childhood-onset UC, higher admissions rates for acute severe exacerbations along with higher colectomy rates have been reported in the paediatric population (5, 6). In children, 15–30% of those with UC experience an acute severe attack at some point (7). Other considerations that are unique to the paediatric population include disease and treatment impacts on growth, pubertal development, bone health, educational impact, and psychosocial wellbeing, making timely optimal treatment a priority.

The paediatric ulcerative colitis activity index (PUCAI) is a validated scoring system used to assess the severity of paediatric UC that is incorporated in the joint ECCO and ESPGHAN guidelines for management of paediatric UC (8–10). The index includes six variables (abdominal pain, rectal bleeding, stool consistency, frequency of stools, nocturnal stools, and limitation of activity) that are individually scored to give a total PUCAI of between 0 and 85. Mild disease is defined as a PUCAI of 10–35, moderate disease as PUCAI of 40–60, and severe disease as a PUCAI of 65 or above.

Acute severe ulcerative colitis (ASC) is considered a medical emergency requiring immediate management, in order to prevent complications such as intestinal perforation, peritonitis, sepsis, and even death. Since the 1950s, intravenous corticosteroid (IVCS) therapy has been shown to reduce mortality in ASC and has become the mainstay of treatment (11). Steroid-refractory cases make up a significant proportion of the paediatric population, as it is estimated to occur in one-third of ASC cases (12). Recently, published data on a large cohort of children with treatment naive UC, who were initially managed with IVCS, showed remission rates of 40%, need for anti-TNF treatment in 24% and a 3% colectomy rate at 4 weeks post-commencement of treatment. Strong predictors for the need for additional medical treatment or colectomy in this same group of children included, high-total Mayo clinical and endoscopic severity score, decreasing serum albumin, rectal biopsy eosinophil count, and rectal biopsy surface viliform changes (13). Effectiveness of first-line treatment with IVCS can be measured using PUCAI on day 3 and day 5 of treatment (14). At day 3 of treatment, a PUCAI of >45 is considered indicative of steroid-refractory disease and at this time second-line treatment options should be considered (10). Colectomy was historically the second-line treatment option for steroid-refractory ASC in many centres. While this may still be appropriate in certain cases, current European guidelines also propose the use of second-line medical management with agents such as infliximab, tacrolimus, and cyclosporine (10).

Infliximab is a chimeric monoclonal antibody to human TNF-α, which has been shown to be effective in modulating intestinal inflammation in UC (15). A landmark prospective study of paediatric ASC reported reduced colectomy rates in patients receiving infliximab by discharge (9%) and 1-year follow-up (19%) (14). The use of infliximab in children with ASC has been reported in six-case series, with a pooled short-term response rate of 75% and a long-term response rate of 64% (12, 14, 16). Infliximab was first introduced for the treatment of ASC in children at Our Lady’s Children’s Hospital, Crumlin (OLCHC) in 2011. The aims of this study were to determine the outcomes of Irish children with severe UC; the impact of infliximab on these outcomes following its introduction; and the outcomes of infliximab therapy in children with UC since its introduction.

## Materials and Methods

The study was carried out at the National Centre for Paediatric Gastroenterology, OLCHC, Dublin, which is the only tertiary referral centre for paediatric gastroenterology in the Republic of Ireland. A retrospective chart review was carried out of all patients admitted with an episode of ASC between January 1, 2009 and December 31, 2015. Patients were identified using the hospital discharge coding system and the department’s patient database. Patients were eligible for inclusion in the study if they had a new or pre-existing diagnosis of UC *and* required acute admission *and* had a PUCAI score of ≥65 and/or a physician global assessment rating of severe disease activity. Although the PUCAI score was still being developed at the beginning of the study period, the elements of the score were being recorded as part of standard clinical care of the time.

Patients were phenotyped according to the Paris classification (17) following diagnostic work up according to the Porto criteria (18). Data collected included gender, age at diagnosis of UC, age at episode of ASC, and endoscopic disease location and histological findings. PUCAI scores on days 3, 5 and at discharge were also recorded along with laboratory values including CRP, ESR, and albumin values. Clinical outcomes recorded included remission (defined as PUCAI ≤10), colectomy, adjunctive medications, adverse treatment events, and post-colectomy complications.

A subgroup analysis compared outcomes in patients treated prior to and after the introduction of infliximab at OLCHC. The standard induction protocol with infliximab for ASC involved doses of 5 mg/kg at weeks 0, 2, and 6 (9, 10). Standard maintenance protocol involved doses of 5 mg/kg every 8 weeks from week 14. Depending on clinical response, modifications to the regimen were made at clinician discretion accordingly. Patients were considered to have undergone accelerated dosage when either infliximab dose/kilogram was increased and/or if infliximab was administered sooner than outlined in the standard protocol. Outcomes of all UC cases managed with infliximab were analysed including clinical response and progression to colectomy.

Statistical analysis was carried out using IBM SPSS Statistics version 24 (IBM, Armonk, NY, USA). Continuous variables were analysed using Mann–Whitney or *t*-test and categorical variables analysed using χ^2^ test or Fisher’s exact test. Kaplan–Meier survival curve was used to show the colectomy free interval from infliximab commencement. Multivariate logistic regression was used to evaluate the associations between remission and predictor factors.

## Results

A total of 55 patients with an episode of ASC were identified over the 7-year study period with a median follow-up of 29 months (IQR, 16.5–48.5). Patient characteristics are described in Table 1. Females accounted for 58% (32/55) of the population. The average age at episode of ASC was 11.7 years (SD ± 3.9 years), with an average age at diagnosis of UC of 11.1 years (SD ± 3.7 years). Endoscopic disease location was documented as left-sided colitis in 2% (1/55), extensive colitis in 7% (4/55), and pan colitis was present in 91% (50/55) of cases. Extra-intestinal manifestations of IBD were present in 6% (3/55) of cases. Steroid-dependent disease was reported in 40% (22/55). Prior to commencement of treatment for ASC, 64% (35/55) of the patients were on maintenance medical therapy, including oral 5-ASA’s (62%; 34/55), rectal 5-ASA’s (24%; 13/55), and oral steroids (46%; 25/55). A further 5% (*n* = 3) subsequently commenced adalimumab. The mean duration of IVCS treatment was 7.3 days (SD ± 3.8 days), with average doses equivalent to 2.3 mg/kg/day of methylprednisolone. The mean PUCAI scores on day 3 and day 5 IVCS were 57 (SD ± 15.1) and 51 (SD ± 20.2), respectively.

**Table 1:** Characteristics of patients with ASC.

Total patient number	*N* = 55
Male: female	1:1.4

Average age at ASC	11.8 years (SD ± 3.9)

Average age at diagnosis of UC	11.1 years (SD ± 3.7)

Endoscopic disease location	
– Left-sided colitis	2% (*n* = 1)
– Extensive colitis	7% (*n* = 4)
– Pancolitis	91% (*n* = 50)

Extra-intestinal manifestations of IBD	6% (*n* = 3)

Treatment at time of admission	(*n* = 35)
– Topical/rectal 5-ASA	24% (*n* = 13)
– Oral steroids	46% (*n* = 25)
– Oral 5-ASA	62% (*n* = 34)

PUCAI score:	
– Day 3 of IVCS	57 (SD ± 15.1)
– Day 5 of IVCS	51 (SD ± 20.2)

Colectomy	(*n* = 22)
– Acute/urgent setting	20% (*n* = 11)
– Elective/semi-elective	20% (*n* = 11)

Remission status at time of maximal follow-up	56% (*n* = 29)

*ASC, acute severe ulcerative colitis; IBD, inflammatory bowel disease; IVCS, intravenous corticosteroids; UC, ulcerative colitis*.

Thirty-three patients were subsequently treated with infliximab as second-line medical therapy, with 18/33 (55%) receiving our standard induction protocol. The remaining patients had protocol modifications in response to their clinical status, including increased dosage and reduced intervals between infusions. Most patients (69%; 23/33) subsequently required an increase in dose to 10 mg/kg. The mean number of infusions before dose escalation was four doses (SD ± 2.8 doses). A similar proportion, 64% (21/33), required a reduction in interval time between infusions. Fifteen patients required escalation prior to week 14 (i.e., during the induction period). Six of these 15 patients ultimately needed a colectomy within a median time of 2.6 months (IQR, 1.2–2.9), 6 in remission as at maximum follow-up, 1 patient switched to adalimumab, and the remainder had some degree of ongoing disease activity. Conversely, 18 patients completed the standard induction, 3 of whom eventually had a colectomy within 2.6 months (IQR, 2.3–20.5) and 14 of whom reached remission on infliximab. The mean number of days from infliximab commencement to dose escalation in the 23 patients was 111 days (SD ± 130.2 days). Of the 23 patients, 52% (12/23) were in remission as at last date of follow-up and 30% (7/23) had required a colectomy. The mean duration of time from diagnosis of UC to starting infliximab was 9.8 months (SD ± 12.5 months) and the mean duration of subsequent infliximab therapy was 2.4 years (SD ± 1.1 years) from the first infusion.

There was an apparent increase in the mean annual number of cases of ASC from 2011, from 4.0 to 9.4 cases per year, in tandem with the increased incidence of IBD in the Irish population, as previously reported (19).

**Figure 1:**
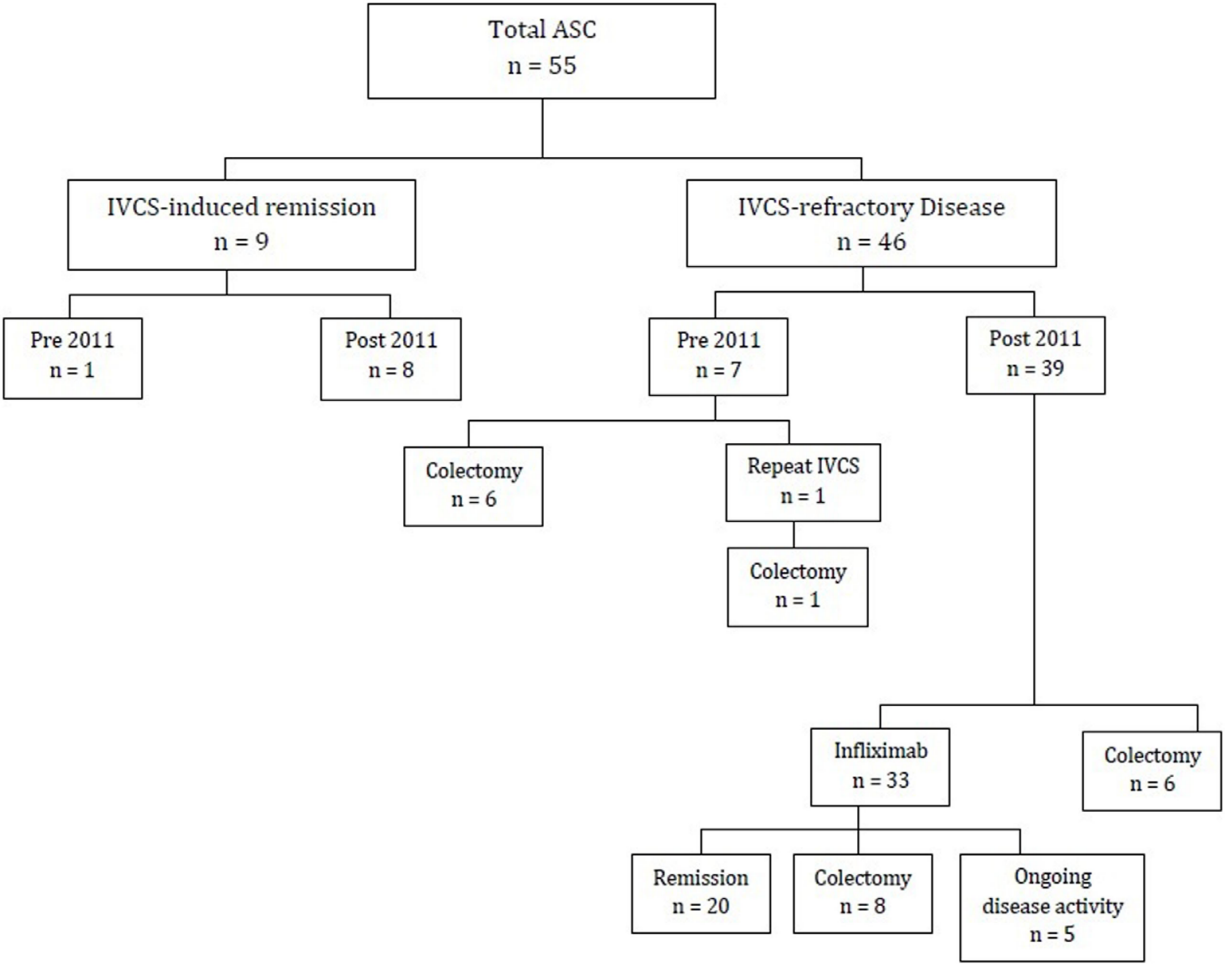
Flow diagram of patients diagnosed with ASC (2009–2015). ASC, acute severe ulcerative colitis; IVCS, intravenous corticosteroids.

Following first-line treatment with IVCS, only 16% (9/55) of the study population achieved remission. The remaining 86% (46/55) required further treatment in the form of either repeat course of IVCS, colectomy, or infliximab. These were divided into two groups: those managed prior to the introduction of infliximab in 2011 (*n* = 7) and those managed from 2011 onwards (*n* = 39). Of those treated prior to 2011, 86% (6/7) had a colectomy as second-line treatment. The remaining patient had a second course of IVCS but ultimately required a colectomy. In those treated from 2011 onwards, 15% (6/39) underwent colectomy as second-line therapy (including cases whereby parents declined infliximab), while the remaining 85% (33/39) were commenced on infliximab. Of the 47 patients managed after 2011, 14 underwent a colectomy. Figure 1 gives a breakdown of reasons necessitating colectomies.

Twenty of 33 patients on infliximab (60%) entered remission following induction, 8 (24%) underwent a colectomy within a median time of 1.9 months (IQR, 3–5) from the commencement of infliximab. Of the remaining five patients, two lost infliximab response and switched to adalimumab and three remained on infliximab without fully achieving remission. Of the 20 patients who entered remission on infliximab, 80% (16/20) were still on infliximab by the end of the study period, 15% (3/20) were successfully weaned off infliximab following prolonged sustained remission and one patient discontinued infliximab due to severe periorbital cellulitis subsequently required a colectomy. Nine patients were commenced on thiopurines after starting infliximab. Of the total 33 patients on infliximab, only 3 patients had adverse events, 1 episode of flushing and acute desaturations, which recovered, 1 liver injury and 1 severe periorbital cellulitis. The latter two patients required discontinuation of infliximab.

**Figure 2:**
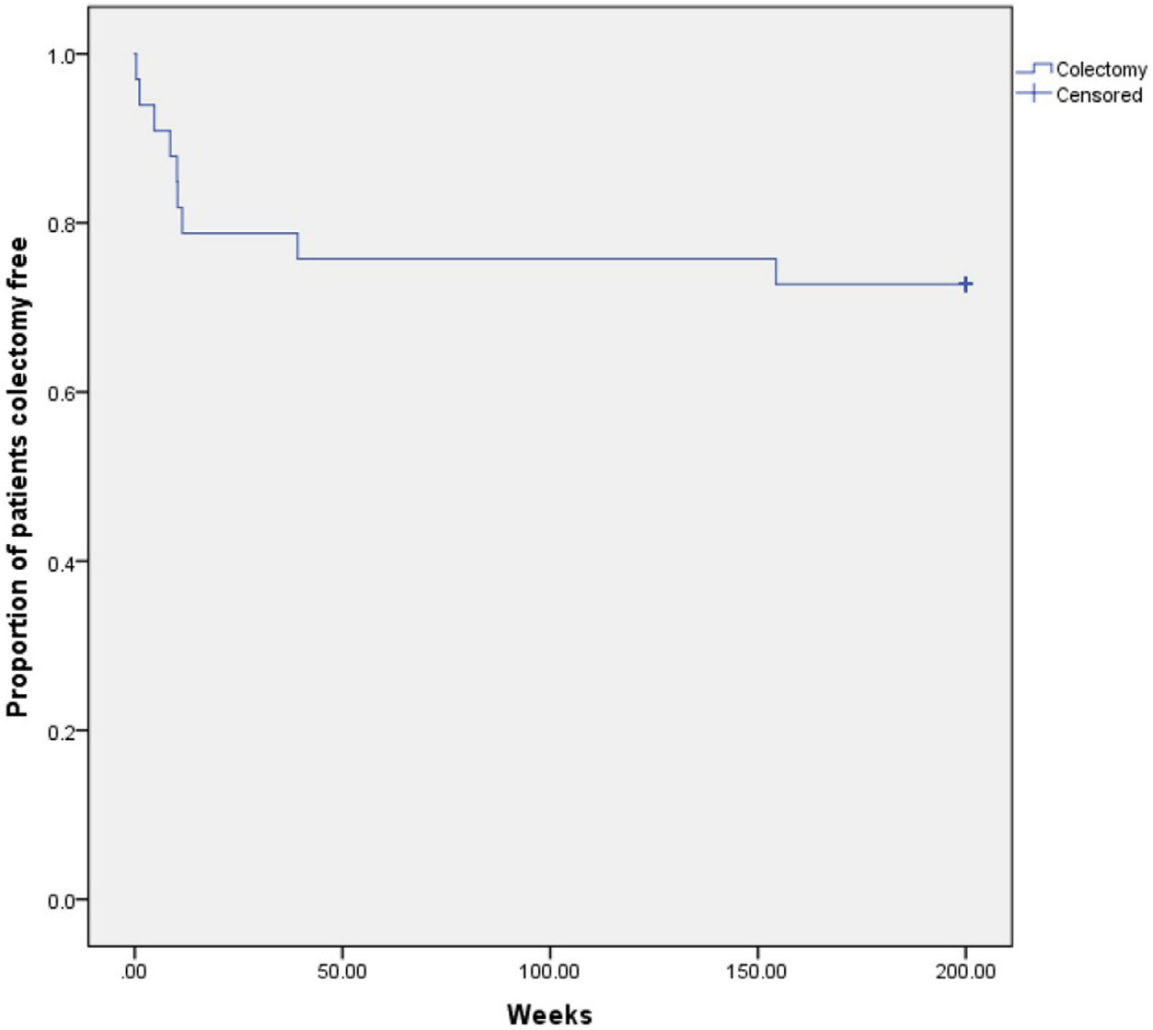
Kaplan–Meier cumulative time to colectomy for infliximab patients.

There were 14 patients treated after 2011 who required a colectomy, 6 as second-line treatment for steroid-refractory ASC and 8 as third-line management, following treatment failure on infliximab. Gender, age at diagnosis, CRP, PUCAI on days 3 and 5, along with receiving standard induction protocol did not significantly increase the odds of a colectomy (Table 2). Kaplan–Meier survival estimates of the cumulative probability for colectomy in patients treated with infliximab were 24% at 1 year (Figure 2), with 50% undergoing colectomy by week 10 following treatment with infliximab. The 20 patients who achieved remission on infliximab were further examined in order to identify any variables, which may predict successful remission. The variables of gender, age at diagnosis, CRP, PUCAI on days 3 and 5, receipt of standard induction protocol, and first episode of ASC were all analysed. None of these variables showed a statistical significance in predicting outcome (Table 3).

**Table 2:** Predictive factors for requiring a colectomy post 2011.

Variable	Odds ratio	CI	*P*-value
Gender	1.1	0.016–73.868	0.965
Age at diagnosis	1.3	0.637–2.442	0.520
CRP	1.0	0.986–1.045	0.318
PUCAI D3	1.0	0.844–1.11	0.646
PUCAI D5	0.3	0.907–1.374	0.300
Standard induction	0.1	0.002–2.642	0.152

*CRP, C reactive protein; PUCAI, paediatric ulcerative colitis activity index*.

**Table 3:** Predictive factors for achieving remission on infliximab.

Variable	Odds ratio	CI	*P*-value
Gender	4.381	0.273–70.189	0.297
Age at diagnosis	0.813	0.536–1.235	0.332
CRP	0.993	0.971–1.015	0.510
PUCAI D3	1.095	0.962–1.248	0.171
PUCAI D5	0.911	0.805–1.03	0.137
Standard induction	6.355	0.479–84.347	0.161
First episode of ASC	0.311	0.009–10.722	0.518

*CRP, C reactive protein; PUCAI, paediatric ulcerative colitis activity index; ASC, acute severe ulcerative colitis*.

Age of disease onset, based on the Paris classification, did not have a significant impact on the outcome of infliximab therapy in our group. Most infliximab-treated patients were over the age of 10 years at diagnosis (79%; 26/33). There were fewer girls aged less than 10 years at diagnosis (*n* = 3), but fewer boys (*n* = 7) aged over 10 at diagnosis and the mean age of diagnosis was 7 years (SD ± 2.5 years) and 14 years (SD ± 1.6 years) in the respective groups.

## Discussion

This is the first report of the outcomes of ASC in the Irish paediatric population, spanning the time immediately before and since the introduction of infliximab as second-line therapy for ASC. This was a single-centre retrospective cohort study, but as Ireland has a single paediatric IBD centre, the data are representative of the Irish population. In this study, the overall colectomy rate of 40% is in keeping with data from studies of a similar era. A 2007 Canadian study reported colectomy rates at discharge, 1 and 6 years of 42, 58, and 61%, respectively (5). Since the introduction of infliximab in the study centre for ASC rescue therapy, the colectomy rate has declined compared with the preceding epoch, although the periods were neither of equivalent time nor patient number. Previous adult and paediatric studies have shown infliximab to significantly reduce colectomy rates in ASC, reducing surgical rates to 19% at 1-year follow-up in one large paediatric study (14, 20, 21). The impact of infliximab rescue therapy on longer-term outcomes of ASC is less clear. Data from this current study are limited in this regard, but the long-term colectomy rate in published studies remains considerable (13, 22).

The clinical response to infliximab therapy in this study was broadly in keeping with previously published reports, with 70% of patients showing sustained clinical response. A 2011 systematic review showed a 1-year response rate of 64% (12). Other studies have demonstrated long-term response rates from 57 to 61% (16, 23) in paediatric UC. In contrast, data from this study also suggest a less favourable response to steroids in Irish children with ASC. The reasons for this are not clear from this particular study, but are under investigation in an ongoing prospective study of paediatric IBD in Ireland. While patient numbers are small, even the IVCS response rates in the infliximab era are less than reported in previous studies (5). The mean day 3 PUCAI scores of patients requiring second-line therapy in this study were consistent with prediction models previously published by Turner et al. (8). The impact of international paediatric ASC management guidelines on reported disease course and outcomes in this study is difficult to objectively quantify, but they have undoubtedly influenced the reported natural history of ASC in the immediate hospitalisation period. Evidence-based guidelines now advocate prompt planning and initiation of second-line therapy, guided by PUCAI score dynamics. It is no longer conscionable that children with unresponsive ASC, continue on indefinite IVCS therapy, as may have happened in the pre-infliximab era, and IVCS-refractory ASC is now diagnosed within days rather than weeks of admission.

This study includes the earliest patients with ASC in our centre that had protocol acceleration of infliximab treatment for UC. Recent reports have described potential benefits of accelerated infliximab induction regimens for optimal disease control and reducing the early colectomy rate in severe UC (24, 25). Shapiro et al. suggest that an initial intensive regimen is necessary in extensive disease (90% of the patients in this study) to maintain sustained efficacy (26). Therapeutic drug monitoring may be a useful adjunct to clinical judgment to identify which patients need therapy escalation with infliximab (27). In the current study, such monitoring was not available across the entire study period so we were unable to reach conclusions in this regard.

This study is a descriptive account of the outcomes of ASC in our national paediatric cohort over a 7-year period, but substantive extrapolation is tempered by the limitations of its retrospective design. It was not possible to extend the study before 2009 due to our resource limitations. The resulting small patient numbers and inequitable “era” groups curtailed the generation of more robust statistical findings and comparative analysis. The study period coincided with a significant and sustained increase in the incidence of paediatric IBD in Ireland, which may account in part for the increased occurrence of ASC over time (19, 28). The advent of the PUCAI score and published guidelines for modern management of ASC also evolved during the study, adding to the potential heterogeneity of the clinical management observed. Small case numbers may account for the seemingly disproportionate numbers of IVCS refractory disease in our population. However, IVCS refractory ASC was substantial across the entire time period and objective measures including day 3 PUCAI scores were consistent with the published literature regarding need for second-line therapy. It is tempting to speculate that delayed access or delayed presentation of children from areas remote to our hospital may have contributed to disease burden by the time of their admission for IVCS treatment, but elucidating these data were not possible retrospectively.

This is the first study of paediatric ASC in the Irish population. It provides further supportive evidence of the beneficial impact of infliximab on the otherwise natural history of this condition in children, although a considerable proportion of children required escalation of infliximab therapy beyond a standard induction regimen. More robust prediction models for early identification of patients at risk of steroid unresponsiveness or needing infliximab escalation are keenly awaited from ongoing collaborative prospective studies.

## Ethics Statement

The Research Ethics Committee of Our Lady’s Children’s Hospital, Crumlin approved the DOCHAS study. The committee does not require prior patient consent for endeavours that only involve retrospective chart reviews undertaken for the purpose of anonymised clinical data collection, audit, and analysis. The current study falls into the latter category.

## Author Contributions

AA, TR, RO, and SH contributed to the conception and design of this work along with the statistical analysis of this work. All authors contributed to the generation and organisation of the database. AA, RO, and SH wrote sections of the manuscript. All authors have read and approved the submitted version.

## Conflict of Interest Statement

The authors declare that the research was conducted in the absence of any commercial or financial relationships that could be construed as a potential conflict of interest.

## References

[1] Henderson P Wilson DC The rising incidence of paediatric onset inflammatory bowel disease. Arch Dis Child (2012) 97:585–6. 10.1136/archdischild-2012-302018 22745290

[2] Ungaro R Mehandru S Allen PB Peyrin-Biroulet L Colombel JF. Ulcerative colitis. Lancet (2017) 389:1756–70. 10.1016/S0140-6736(16)32126-2 27914657 PMC6487890

[3] Ng SC Shi HY Hamidi N Underwood FE Tang W Benchimol EI Worldwide incidence and prevalence of inflammatory bowel disease in the 21st century: a systematic review of population-based studies. Lancet (2018) 390:2769–78. 10.1016/S0140-6736(17)32448-0 29050646

[4] Benchimol EI Fortinsky KJ Gozdyra P Van den Heuvel M Van Limbergen J Griffiths AM. Epidemiology of pediatric inflammatory bowel disease: a systematic review of international trends. Inflamm Bowel Dis (2011) 17:423–39. 10.1002/ibd.21349 20564651

[5] Turner D Walsh CM Benchimol EI Mann EH Thomas KE Chow C Severe paediatric ulcerative colitis: incidence, outcomes and optimal timing for second-line therapy. Gut (2008) 57:331–8. 10.1136/gut.2007.136481 17981888

[6] Gower-Rousseau C Dauchet L Vernier-Massouille G Tilloy E Brazier F Merle V The natural history of pediatric ulcerative colitis: a population based cohort study. Am J Gastroenterol (2009) 104(8):2080–8. 10.1038/ajg.2009.177 19436273

[7] Romano C Syed S Valenti S Kugathasan S. Management of acute severe colitis in children with ulcerative colitis in the biologics era. Pediatrics (2016) 137:5. 10.1542/peds.2015-1184 27244779

[8] Turner D Hyams J Markowitz J Lerer T Mack DR Evans J Appraisal of the pediatric ulcerative colitis activity index (PUCAI). Inflamm Bowel Dis (2009) 15:1218–23. 10.1002/ibd.20867 19161178

[9] Turner D Levine A Escher JC Griffiths AM Russell RK Dignass A Management of pediatric ulcerative colitis: joint ECCO and ESPGHAN evidence-based consensus guidelines. J Pediatr Gastroenterol Nutr (2012) 55:340–61. 10.1097/MPG.0b013e3182662233 22773060

[10] Turner D Travis SP Griffiths AM Ruemmele FM Levine A Benchimol EI Consensus for managing acute severe ulcerative colitis in children: a systematic review and joint statement from ECCO, ESPGHAN, and the Porto IBD Working Group of ESPGHAN. Am J Gastroenterol (2011) 106:574–88. 10.1038/ajg.2010.481 21224839

[11] Truelove SC Witts LJ Cortisone in ulcerative colitis; final report on a therapeutic trial. Br Med J (1955) 29(2):1041–8. 10.1136/bmj.2.4947.1041 PMC1981500 13260656

[12] Turner D Griffiths AM. Acute severe ulcerative colitis in children: a systematic review. Inflamm Bowel Dis (2011) 17(1):440–9. 10.1002/ibd.21383 20645317

[13] Hyams JS Davis S Mack DR Boyle B Griffiths AM LeLeiko NS Factors associated with early outcomes following standardised therapy in children with ulcerative colitis (PROTECT): a multicentre inception cohort study. Lancet Gastroenterol Hepatol (2017) 2(12):855–68. 10.1016/S2468-1253(17)30252-2 28939374 PMC5695708

[14] Turner D Mack D Leleiko N Walters TD Uusoue K Leach ST Severe pediatric ulcerative colitis: a prospective multicenter study of outcomes and predictors of response. Gastroenterology (2010) 138(7):2282–91. 10.1053/j.gastro.2010.02.047 20193683

[15] Sandborn WJ Hanauer SB. Antitumor necrosis factor therapy for inflammatory bowel disease: a review of agents, pharmacology, clinical results, and safety. Inflamm Bowel Dis (1999) 5(2):119–33. 10.1097/00054725-199905000-00008 10338381

[16] Hyams JS Lerer T Griffiths A Pfefferkorn M Stephens M Evans J Outcome following infliximab therapy in children with ulcerative colitis. Am J Gastroenterol (2010) 105(6):1430–6. 10.1038/ajg.2009.759 20104217

[17] Levine A Griffiths A Markowitz J Wilson DC Turner D Russell RK Pediatric modification of the Montreal classification for inflammatory bowel disease: the Paris classification. Inflamm Bowel Dis (2011) 17(6):1314–21. 10.1002/ibd.21493 21560194

[18] Levine A Koletzko S Turner D Escher JC Cucchiara S de Ridder L ESPGHAN revised Porto criteria for the diagnosis of inflammatory bowel disease in children and adolescents. J Pediatr Gastroenterol Nutr (2014) 58(6):795–801. 10.1097/MPG.0000000000000239 24231644

[19] Hope B Shahdadpuri R Dunne C Broderick AM Grant T Hamzawi M Rapid rise in incidence of Irish paediatric inflammatory bowel disease. Arch Dis Child (2012) 97(7):590–4. 10.1136/archdischild-2011-300651 22550323

[20] Järnerot G Hertervig E Friis-Liby I Blomquist L Karlén P Grännö C Infliximab as rescue therapy in severe to moderately severe ulcerative colitis: a randomized, placebo-controlled study. Gastroenterology (2005) 128(7):1805–11. 10.1053/j.gastro.2005.03.003 15940615

[21] Florholmen J Øverland G Olsen T Rismo R Cui G Christiansen I Short-and long-term clinical outcomes of infliximab in fulminant ulcerative colitis. Ulcers (2011) 2011:7 10.1155/2011/156407

[22] Dayan B Turner D. Role of surgery in severe ulcerative colitis in the era of medical rescue therapy. World J Gastroenterol (2012) 18:3833–8. 10.3748/wjg.v18.i29.3833 22876035 PMC3413055

[23] Iwańczak BM Kierkuś J Ryżko J Szczepanik M Więcek S Czaja-Bulsa G Induction and maintenance infliximab therapy in children with moderate to severe ulcerative colitis: retrospective, multicenter study. Adv Clin Exp Med (2017) 26(1):57–61. 10.17219/acem/42197 28397433

[24] Gibson DJ Heetun ZS Redmond CE Nanda KS Keegan D Byrne K An accelerated infliximab induction regimen reduces the need for early colectomy in patients with acute severe ulcerative colitis. Clin Gastroenterol Hepatol (2015) 13(2):330–5. 10.1016/j.cgh.2014.07.041 25086187

[25] Hindryckx P Novak G Vande Casteele N Laukens D Parker C Shackelton LM Review article: dose optimisation of infliximab for acute severe ulcerative colitis. Aliment Pharmacol Ther (2017) 45(5):617–30. 10.1111/apt.13913 28074618 PMC6658182

[26] Shapiro JM Subedi S Machan JT Cerezo CS Ross AM Shalon LB Durability of infliximab is associated with disease extent in children with inflammatory bowel disease. J Pediatr Gastroenterol Nutr (2016) 62(2):867–72. 10.1097/MPG.0000000000001034 26583483

[27] Kelly OB Donnell SO Stempak JM Steinhart AH Silverberg MS. Therapeutic drug monitoring to guide infliximab dose adjustment is associated with better endoscopic outcomes than clinical decision making alone in active inflammatory bowel disease. Inflamm Bowel Dis (2017) 23(7):1202–9. 10.1097/MIB.0000000000001126 28498155

[28] Coughlan A Wylde R Lafferty L Quinn S Broderick A Bourke B A rising incidence and poorer male outcomes characterise early onset paediatric inflammatory bowel disease. Aliment Pharmacol Ther (2017) 45(12):1534–41. 10.1111/apt.14070 28449214

